# Supplementary material for: Molecular characterization of Salmonella spp. and Listeria monocytogenes strains from biofilms in cattle and poultry slaughterhouses located in the federal District and State of Goiás, Brazil
Source: PLoS One. 2021 Nov 12;16(11):e0259687. doi: 10.1371/journal.pone.0259687 (PMC8589217; doi:10.1371/journal.pone.0259687)
Supplement: S1 Fig — (PDF) [file pone.0259687.s001.pdf]

Original raw gel images used to support results presented in **Figure 1**. Gel 3 was a replication of the first half of gel 1 for better visibility of band patterns.

Gel 1:

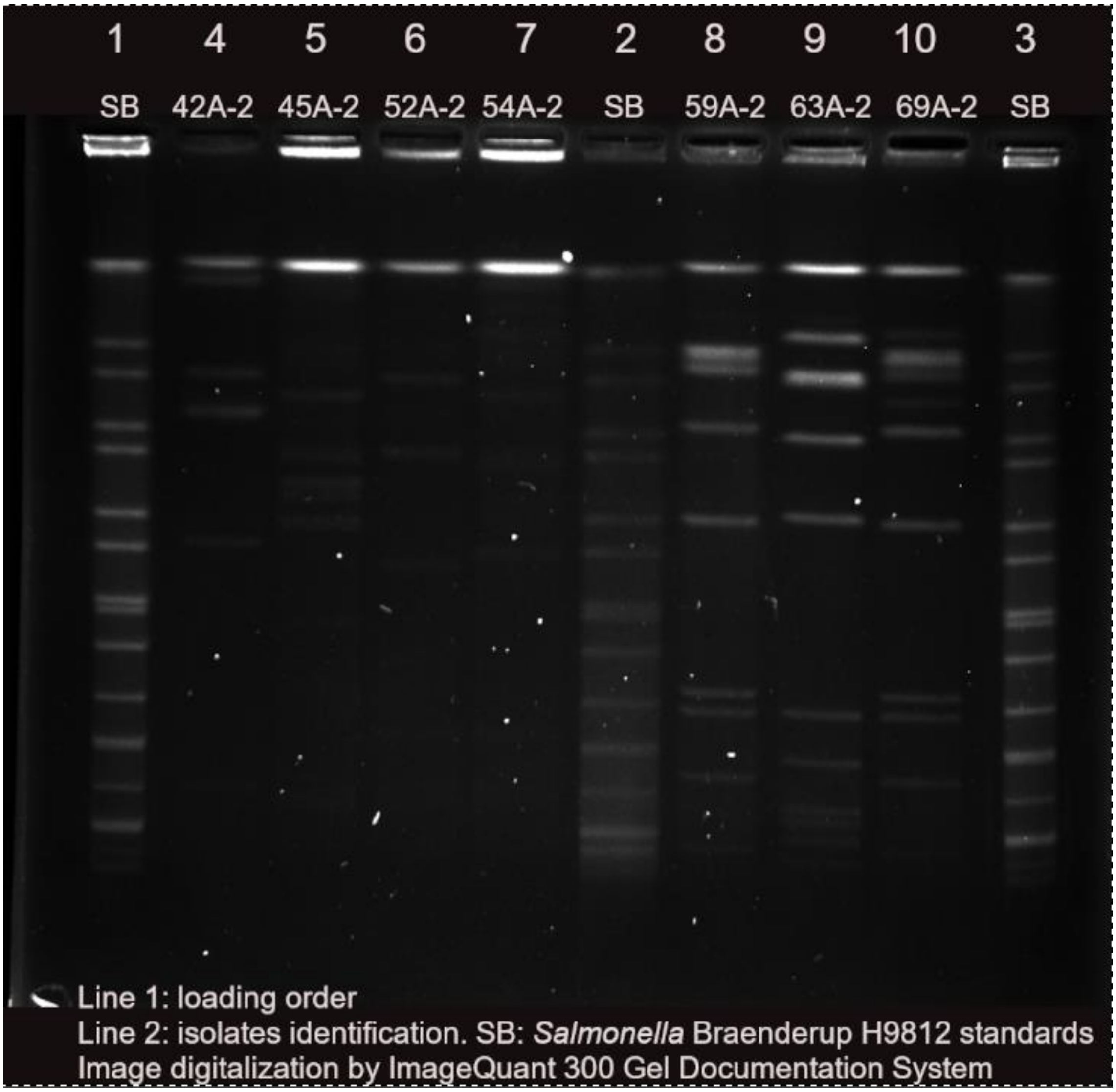

Gel 2:

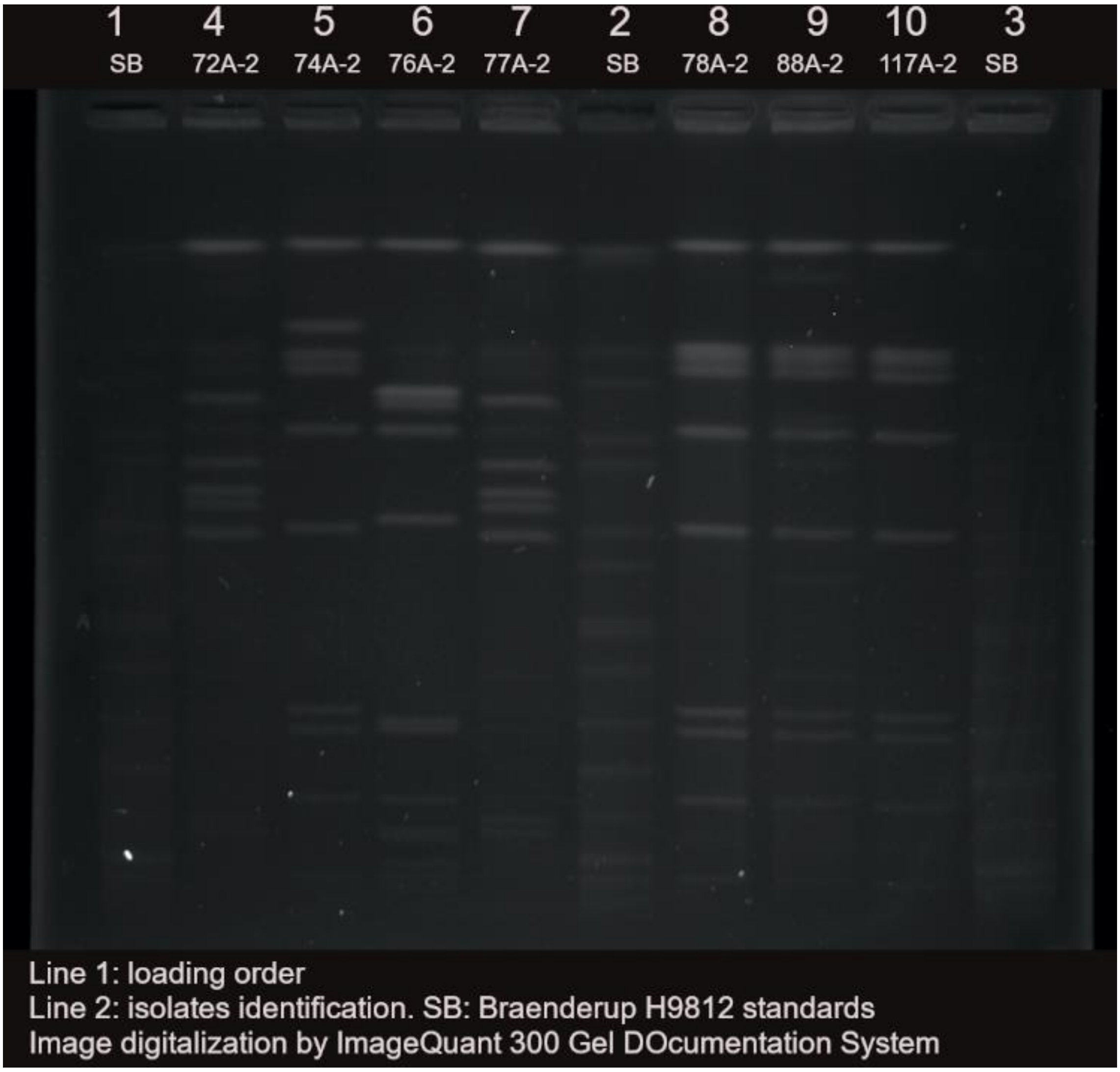
1 4 5 6 7 2 8 9 10 3
SB 72A-2 74A-2 76A-2 77A-2 SB 78A-2 88A-2 117A-2 SB
Line 1: loading order
Line 2: isolates identification. SB: Braenderup H9812 standards
Image digitalization by ImageQuant 300 Gel DOcumentation System

Gel 3:

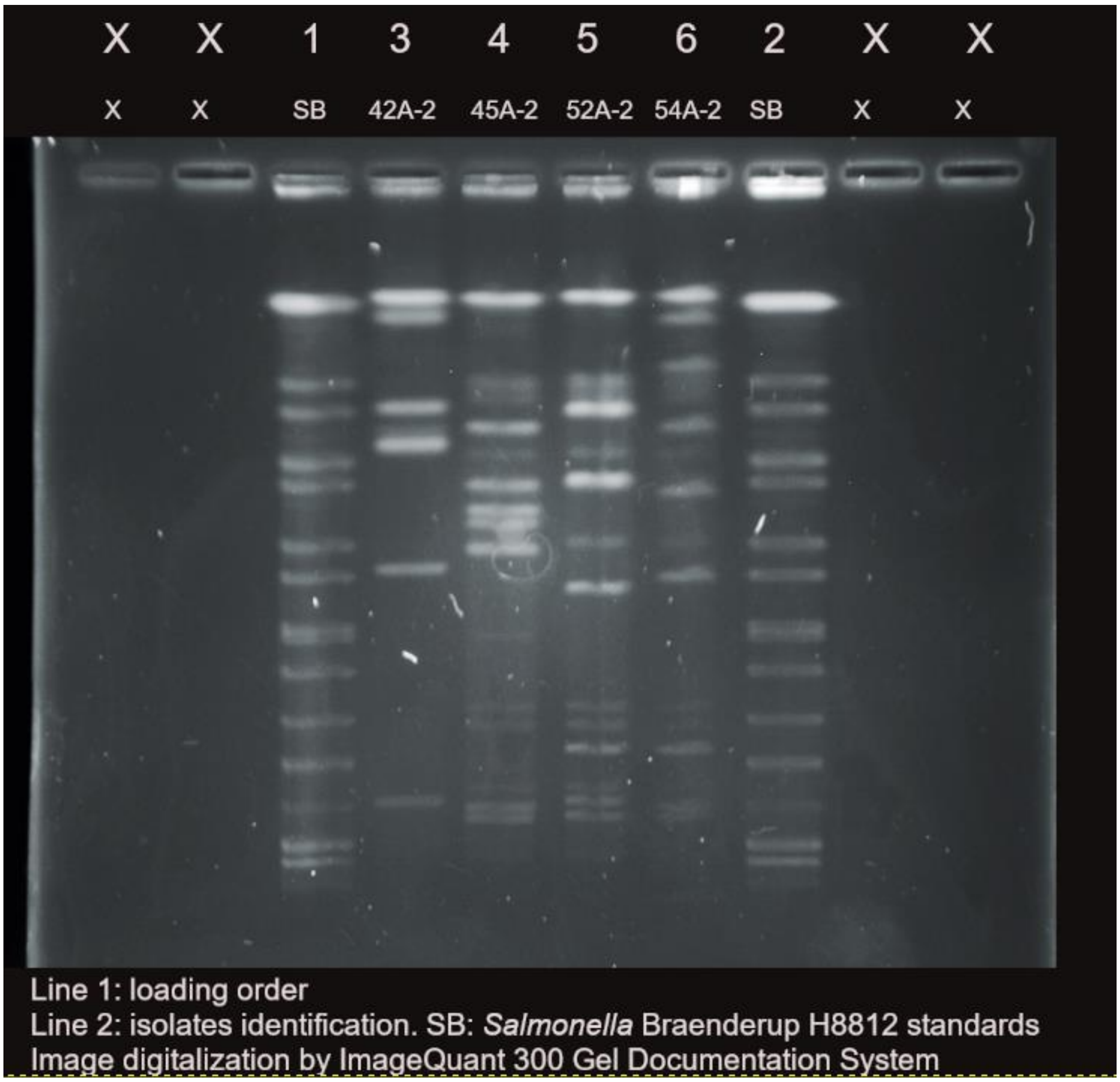

Line 1: loading order
Line 2: isolates identification. SB: *Salmonella* Braenderup H8812 standards
Image digitalization by ImageQuant 300 Gel Documentation System
