# Supplementary material for: Molecular characterization of Salmonella spp. and Listeria monocytogenes strains from biofilms in cattle and poultry slaughterhouses located in the federal District and State of Goiás, Brazil
Source: PLoS One. 2021 Nov 12;16(11):e0259687. doi: 10.1371/journal.pone.0259687 (PMC8589217; doi:10.1371/journal.pone.0259687)
Supplement: Attachment — Submitted filename: Manuscript_tracked changes.rtf [file pone.0259687.s002.rtf]

Molecular characterization of Salmonella spp. and Listeria monocytogenes strains from biofilms in cattle and poultry slaughterhouses located in the Federal District and State of Goiás, Brazil

Emilia Fernanda Agostinho Davanzo^1¶*^, Rebecca Lavarini dos Santos^1&^, Virgílio Hipólito de Lemos Castro^1&^, Joana Marchesini Palma^1&^, Bruno Rocha Pribul^2&^, Bruno Stéfano Lima Dallago^1&^, Bruna Fuga^3&^, Margareti Medeiros^1&^, Simoneide Souza Titze de Almeida^1&^, Hayanna Maria Boaventura da Costa^1&^, Dália dos Prazeres Rodrigues^2&^, Nilton Lincopan^3&^, Simone Perecmanis^1&^, Angela Patrícia Santana^1¶^. 

^1^ Faculty of Agronomy and Veterinary Medicine, University of Brasília (UnB), Brasília - DF, Brazil.
^2^ National Reference Laboratory for Bacterial Enteric Infections, Oswaldo Cruz Institute, Manguinhos, Rio de Janeiro - RJ, Brazil.
^3^ Laboratory of Bacterial Resistance and Therapeutic Alternatives, Biomedical Sciences Institute, University of São Paulo, São Paulo - SP, Brazil. 

*Corresponding author
E-mail: emiliadavanzo@unb.br (EFAD)

¶These authors contributed equally to this work.
&These authors also contributed equally to this work.


Abstract
	
Listeria monocytogenes and Salmonella are important foodborne pathogens and are commonly associated with foods of animal origin, according to the Centers for Disease Control and Prevention. The aim of this work was to perform molecular characterization of L. monocytogenes and Salmonella spp. strains isolated from biofilms of cattle and poultry slaughterhouses located in the Federal District and Goiás, Brazil. Fourteen L. monocytogenes isolates and one Salmonella spp. isolate were detected in poultry slaughterhouses. No isolates were detected in cattle slaughterhouses. All L. monocytogenes isolates belong to lineage II (serotypes 1/2a, 3a), and 11 different pulsotypes were detected. PFGE analysis revealed the dissemination of two strains within one plant, in addition to the regional dissemination of one of them. The Salmonella isolate was identified by WGS as Salmonella enterica serovar Minesota ST548. In the sequence analysis, no PMSCs were detected in the inlA gene of Listeria. All isolates were able to adhere to Caco-2 cells, while 50% were capable to invade them. Antimicrobial resistance was detected in 57.1% of L. monocytogenes isolates, and sulfonamide resistance was the most observed. The tetC, ermB, and tetM genes were detected and four isolates were classified as multi-drug resistant. Salmonella spp. isolate was resistant to nine antimicrobials and was classified as multidrug-resistant. In addition, were also detected qnrB19, bla_CMY-2_, aac(6')-Iaa, sul2, and tetA resistance genes besides a mutation in the parC gene. The majority (78.5%) of the L. monocytogenes isolates were capable to form biofilms after incubation at 37°C for 24 h and 64.3% were capable to form biofilms after incubation at 12°C for 168 h. There was no statistical difference in the biofilm forming capacity under distinct evaluated conditions. Salmonella spp. isolate was capable to form biofilm at both tested temperatures. Biofilm characterization was confirmed by collecting the samples repetitively, at the same sampling points, and by assessing biofilm formation in vitro. These results highlight the potential risk of cross-contamination in poultry slaughterhouses and the importance of surveillance and pathogen control maintenance programs within the meat production industry.

Keywords: PFGE, WGS, internalin A, antimicrobial resistance

Introduction  

The hygienic-sanitary condition of the industrial environment is an essential factor for food safety and quality guarantee [1–3]. One of the current challenges in slaughtering and meat processing is to avoid product cross-contamination by microorganisms [4,5]. This can occur by contact with bacterial biofilms due to an improper hygiene and sanitization procedure of the slaughter system. This leads to a decrease in food quality resulting in pathogen transmission and in the occurrence of recurrent contamination of produced food [5,6]. Listeria monocytogenes and Salmonella spp. are important pathogens to be controlled in order to guarantee ensure food quality and safety. Their presence is a criteria criterion to be evaluated within the control of meat production process [7].
 L. monocytogenes is the main etiological agent of listeriosis in humans [8]. It is a psychrophilic bacterium, which can develop on surfaces of meat production industries [9]. The species is subdivided into 13 serotypes, from of which 1/2a, 1/2c, and 4b are the most often involved in cases of human listeriosis [10]. Due to the difficulties imposed by traditional serology, different subtyping methods have been developed, such as the differentiation of the main lineages assessed by polymerase chain reaction (PCR) [11]. In addition, molecular typing methods with high discriminatory power, such as pulsed-field gel electrophoresis (PFGE), are widely used to assess clone clonal dissemination within the industries [10,12,13]. Among its clones, the species includes a wide range of pathogenic potential [14]. The role of internalin A in Listeria, encoded by the inlA gene, stands out, as its functionality is necessary for pathogen internalization [15–17]. The presence of premature stop codons (PMSCs) in this gene has been associated with clones with attenuated invasion phenotype [18].
The genus Salmonella, which is responsible for salmonellosis, includes Salmonella enterica species. It is a microorganism often identified as an etiological agent of food outbreaks, and is therefore important for public health, since it causes a disease that is difficult to control and is easily disseminated [19]. These bacteria can adhere to surfaces including metal, glass, and rubber and form biofilms [20–22]. 
Antimicrobial resistance (AMR) is a serious threat to human health [23,24]. To mitigate its risks, the World Health Organization created the Global Action Plan [25]. One of its pillars is to monitor AMR dissemination. Considering the importance of L. monocytogenes and Salmonella spp. for public health, and the few investigations related to the presence of biofilms in Brazil, the objectives of this study were to detect biofilm-induced contamination points and the presence of these microorganisms in processing plants of cattle and poultry slaughterhouses, and to perform the characterization of these strains by serotyping, antimicrobial resistance analysis, and by evaluating the in vitro  biofilm formation capacity of all isolates. In addition, whole genome sequencing (WGS) of Salmonella spp. was performed, besides PFGE, sequencing of inlA gene, and cell adhesion and invasion assays in Caco-2 cells for all Listeria strains.

Material and methods

Sample collection

Samples were collected from three poultry slaughterhouses (A, B and C) and two cattle slaughterhouses (D and E) located in the Federal District area (A, B and D) and in the State of Goiás (C and E). All contained an Official Inspection Service. All the slaughterhouses had agreed to participate voluntarily in this study. Sterile swabs (Absorve) were used to examine the surfaces of facilities, equipment, and utensils of cattle and poultry slaughterhouses, using 25 cm^2^ molds [26]. The collection locations were based on protocols described by Barros et al. 2007 [27] and Nicolau and Bolocan 2014 [26]. The collection points were separated into two groups: installations (floors, walls, and drains of dirty and clean areas); and equipment and utensils (mats, evisceration tables, chutes, different machinery, and utensils like hooks and saws). Sixteen visits were carried out between March 2017 and September 2018, eight in cattle slaughterhouses and eight in poultry slaughterhouses, for 287 collected swabs. Of these, 118 were obtained from cattle slaughterhouses and 169 from poultry slaughterhouses. The swabs were individually added to tubes containing a sterile transport solution (peptone water 0.1%; Himedia) and transported in isothermal boxes to the Food Microbiology Laboratory at the University of Brasília for cultivation and microbiological isolation. Sample processing was done within 24 h of collection. Biofilms were characterized within a processing plant by repetitively collecting samples at the same locations within each plant but on different visits, to observe the recurrence of investigated microorganisms [28]. Studies have correlated the permanence of microorganisms on the surfaces after cleaning and sanitizing to the presence of biofilm on these surfaces [29,30]. Thus, collections were carried out at different times with the locations treated hygienically and sanitized between visits.

Isolation of L. monocytogenes and Salmonella spp.

For isolation of L. monocytogenes, swab samples were analyzed according to methodology described by Ryser and Donnely 2015 [31]. Transport tubes containing swabs were homogenized in a tube shaker, with subsequent addition of 1 mL of transport solution to 9 mL of UMV broth (Acumedia). After 24 h of incubation at 35°C, 0.1 mL of this culture was transferred to 9 mL of Fraser broth (Acumedia). Esculin hydrolysis was done after 24 or 48 h of incubation at 35°C. Each sample was plated on MOX agar (Difco). Suggestive colonies were incubated in BHI broth (Acumedia) for 24 h at 35°C and biochemically analyzed. Samples with characteristics compatible with the genus were examined by PCR to confirm the species using the primers LIP1 [32] and LIP2A [33] for amplification of the prfA gene. PCR was performed as described by Kérouanton et al. 2010 [33]. Positive controls were provided by Dr. Ernesto Hofer of the Oswaldo Cruz Foundation located in Rio de Janeiro.
To identify Salmonella, swab samples were analyzed as described by Ryser and Donnely 2015 [31] and ISO 6579/2002 [34]. After homogenization of transport tubes containing the collected swabs, each homogenate was individually transferred to tubes containing 1% buffered peptone water (Acumedia). Samples were incubated at 37°C for 24 h. Aliquots were transferred to Tetrathionate broth (Merck), Selenite Cystine broth (Merck), and Rappaport-Vassiliadis broth (Merck). After incubation in these selective media, samples were plated on Brilliant Green Phenol Red Agar (Acumedia), Xylose Lysine Deoxycholate (Merck), and Bismuth Sulfite agars (Acumedia). Suggestive colonies were incubated on nutrient agar (Acumedia) and biochemically analyzed using appropriate tests. This was followed by serology with polyvalent anti-Salmonella somatic and flagellar sera (Probac). Genus was confirmed by PCR using OMPCF and OMPCR primers [35] for amplification of the ompC gene, as described by Freitas et al. 2010 [36]. Positive controls were kindly provided by the Oswaldo Cruz Foundation.

Molecular serotyping of L. monocytogenes and Salmonella

L. monocytogenes subtyping used multiplex PCR and lmo0737, lmo1118, ORF2819 and ORF2110 primers as described by Doumith et al. 2004 [11] for isolates differentiation into the four main lineages.
Salmonella spp. serovars were identified by WGS, at the Laboratory of Bacterial Resistance and Therapeutic Alternatives at the Institute of Biomedical Sciences of the University of São Paulo (USP – São Paulo/SP). Genomic DNA was extracted using the PureLink^TM^ Genomic DNA Mini Kit (Life Technologies, CA), and used for library preparation by Nextera DNA Flex Library Prep Kit (Illumina, San Diego, CA). Subsequently, the DNA quantified by Qubit 2.0 fluorometer (Life Technologies) was sequenced on an Illumina NextSeq PE instrument (Illumina Inc., San Diego, CA) using a paired-end (75bp) library. The short reads were trimmed with TrimGalore v0.6.5 (https://github.com/FelixKrueger/TrimGalore) and de novo assembly was performed using the Unicycler v.0.4.8 [37]. Genome annotations were made by NCBI PGAP v.3.2 (http://www.ncbi.nlm.nih.gov/genome/annotation_prok/), and serovar was predicted using SeqSero2 analysis [38].

PFGE of L. monocytogenes

	PFGE was performed as recommended by Centers for Disease Control and Prevention [39]. The restriction enzyme AscI (Invitrogen) was used at 10 U/ìL. As the standard, Salmonella ser. Braenderup H9812 digested with XbaI (Roche) at 10 U/ìL was used, provided by the Enterobacteria Laboratory of the Oswaldo Cruz Foundation.  BioNumerics 7.7. software (Applied Maths) was used to analyze the fragments, with a Dice similarity coefficient at 1.5%. The dendrogram was built by analyzing unweighted pair group method with arithmetic mean (UPGMA) clusters.

Analysis of inlA gene sequence of L. monocytogenes

The sequencing of the inlA gene (2400bp) followed the protocol described by Poyart et al. 1996 [17]. Two gene fragments of 1157 bp and 760 bp were amplified, using O1, O2, O3, and O4 primers [17]. PCR products were purified using the PureLink kit (Invitrogen), quantified by High Mass marker (Invitrogen) and sequenced in ABI3500 sequencer (Applied Biosystems).	

Cell adhesion and invasion assays for L. monocytogenes

	The Caco-2 human colon adenocarcinoma cell line was used. The cells were provided by Prof. Elaine Cristina Pereira De Martinis, from the Faculty of Pharmaceutical Sciences of Ribeirão Preto, University of São Paulo. Cells were cultured as described by Gaillard et al. 1987 [40]. Wells in polypropylene plates (Kasvi) were prepared with a final concentration of 1.0 × 10^5^ cells/well. Adhesion assays were conducted as described by Moroni et al. 2006 [41]. The prepared wells were inoculated with a bacterial suspension using a volume adjusted to obtain a multiplicity of infection (MOI) of 100. After adding Dulbecco's modified Eagles's medium (DMEM) supplemented with 10% fetal bovine serum, the plates were incubated for 2 h in a 5% CO_2_ atmosphere at 37°C. The wells were washed three times with 1× phosphate buffered saline (PBS; Laborclin). The cells were then treated with a lysis buffer solution of 0.1% Triton-X100 (Sigma-Aldrich) and incubated for 10 min using the aforementioned conditions. Viable bacteria were counted by seeding serial dilutions on BHI agar (Difco) and incubating for 24 h at 37°C. The counts were expressed in colony forming units (CFU)/mL. All tests were performed in triplicate. Adhesion of bacteria to Caco-2 cells was calculated (%) as (number of adherent cells × 100) / number of cells adhering to the well [41]. Invasion tests were performed as described by Gaillard et al. 1987 [40] and Moroni et al. 2006 [41]. Cells were added to each well at a MOI of 100. Following incubation, the cells were washed with PBS as described above for the adhesion assay. After the washes, wells were treated with 1 mg/mL of gentamicin (Sigma-Aldrich) and incubated for 1 h in a 5% CO_2_ atmosphere at 37°C. Each well was then treated with the aforementioned lysis buffer solution and viable bacteria were counted as described above for the cell adhesion assay. All tests were performed in triplicate. The percentage of bacterial invasion in Caco-2 cells was determined by the formula: % adhesion = (number of internalized recovered cells × 100) / number of cells adhering to the well.
	
Antibiogram and antimicrobial resistance genes

The antimicrobial resistance of L. monocytogenes isolates was evaluated using the disk diffusion method, as described by the Clinical and Laboratory Standards Institute [42]. The tested antibiotics were ampicillin (10 ìg), ciprofloxacin (5 ìg), chloramphenicol (30 ìg), doxycycline (30 ìg), erythromycin (15 ìg), gentamicin (10 ìg), sulfonamides (300 ìg), and tetracycline (30 ìg). The interpretation of halo diameters was based on standards for Staphylococcus spp. [43] defined by CLSI M100 [42], with the exception of standards for erythromycin and ampicillin, for which the standards defined by the European Committee on Antimicrobial Susceptibility Testing [44] were used for L. monocytogenes. The identification of resistance genes in the L. monocytogenes isolates was performed by PCR. Genes related to resistance to tetracyclines (tetA, tetB, tetC, tetM), macrolides (ermA, ermB, ermC, ereA), amphenicols (cat1, cmlA), sulfonamides (sull), beta-lactams (ampC, blaSHV), and aminoglycosides (aac (3)-I) were investigated. Reactions were carried out in a final volume of 25 ìL, according to the conditions described in studies reported in Table 1 for each pair of primers.

Table 1. Primers used to investigate antimicrobial resistance genes of Listeria monocytogenes isolates.


Gene	Primer	Nucleotide sequence (5'-3')	Size (pb)	Reference	
aac(3)-I	aac(3)-I-F	ACCTACTCCCAACATCAGCC	157	Van et al., 2008	
	aac(3)-I-R	ATATAGATCTCACTACGCGC			
ampC	AmpC-For	TTCTATCAAMACTGGCARCC	550	Schwartz et al., 2003
	
	AmpC-Rev	CCYTTTTATGTACCCAYGA			
bla_SHV_	blaSHV-F	TCGCCTGTGTATTATCTCCC	768	Van et al., 2008	
	blaSHV-R	CGCAGATAAATCACCACAATG			
ermA	ermA-F	TCTAAAAAGCATGTAAAAGAA	645	Sutcliffe et al., 1996	
	ermA-R	CTTCGATAGTTTATTAATATTAGT			
ermB	ermB-F	GAAAAGGTACTCAACCAAATA	639	Sutcliffe et al., 1996	
	ermB-R	AGTAACGGTACTTAAATTGTTTAC			
ermC	ermC-F	TCAAAACATAATATAGATAAA	642	Sutcliffe et al., 1996	
	ermC-R	GCTAATATTGTTTAAATCGTCAAT			
ereA	ere(A)-F	GCCGGTGCTCATGAACTTGAG	419	Van et al., 2008	
	ere(A)-R	CGACTCTATTCGATCAGAGGC			
cat1	CATIF	AGTTGCTCAATGTACCTATAACC	547	Van et al., 2008	
	CATIR	TTGTAATTCATTAAGCATTCTGCC			
cmlA	cmlA-F	CCGCCACGGTGTTGTTGTTATC	698	Van et al., 2008	
	cmlA-R	CACCTTGCCTGCCCATCATTAG			
sul	sull-F	TTCGGCATTCTGAATCTCAC	822	Van et al., 2008	
	sulI-R	ATGATCTAACCCTCGGTCTC			
tetA	tet(A)-F	GTGAAACCCAACATACCCC	887	Van et al., 2008	
	tet(A)-R	GAAGGCAAGCAGGATGTAG			
tetB	tet(B)-F	CCTTATCATGCCAGTCTTGC	773	Van et al., 2008	
	tet(B)-R	ACTGCCGTTTTTTCGCC			
tetC	tet(C)-F	ACTTGGAGCCACTATCGAC	880	Van et al., 2008	
	tet(C)-R	CTACAATCCATGCCAACCC			
tetM	tet(M)-1	GTTAAATAGTGTTCTTGGAG	700	Aarestrup et al., 2000	
	tet(M)-2	CTAAGATATGGCTCTAACAA			


	The antimicrobial resistance of Salmonella spp. was evaluated through the disk diffusion method, according to the CLSI protocol [42]. The tested antibiotics were nalidixic acid (30 µg), amoxicillin (10 µg), ampicillin (10 µg), cephalothin (30 µg), cefazolin (30 µg), ceftazidime (30 µg), ciprofloxacin (5 µg), chloramphenicol (30 µg), colistin (10 µg), doxycycline (30 µg), gentamicin (10 µg), sulfonamides (300 µg), and tetracycline (30 µg). To interpret the results, the standards for Enterobacteriaceae defined by CLSI M100 [42] were used. The presence of resistance genes was evaluated by WGS using ResFinder v.4.0. The presence of genes related to the resistance to the following antimicrobials was investigated: quinolones, tetracycline, nitroimidazole, sulfonamides, macrolides, rifampicin, glycopeptides, colistin, trimethroprim, fusidic acid, aminoglycosides, beta-lactams, oxazolidinone, and fosfomycin. Chromosomal mutations related to antimicrobial resistance in the gyrA, gyrB, pmrA, pmrB, parC, parE, and 16s_rrsD genes were investigated.
	
In vitro biofilm formation capacity 

In vitro biofilm formation capacity of L. monocytogenes and Salmonella spp. isolates was evaluated by incubation at 37°C for 24 h and incubation at 12°C for 168 h (7 days). Tests were performed using polystyrene titration microplates as described by Djordjevic et al. 2002 [45] and modified by Borges et al. 2018 [46]. TSB broth (Acumedia) was used to prepare bacterial suspensions. All tests were performed in triplicate. After preparation, incubation, washing, staining, and resuspension of each sample, the optical density (OD) of each well was measured using an ELx800 enzyme-linked immunosorbent assay reader (Biotek Instruments) at 490 nm. The OD value for each isolate was determined as the arithmetic mean of absorbance readings obtained from the three wells. This value was compared to the value of the negative control (ODn). To determine the capacity and intensity of biofilm formation, the classification proposed by Stepanoviæ et al. 2000 [47] was used. The statistical analysis of the data to compare the capacity of biofilm formation in the two conditions tested was performed using SAS software (SAS Inc.), analyzing the normality by Shapiro-Wilk test followed by the paired t test for means comparison between the two groups.

Results and Discussion

Detection of microorganisms in poultry and cattle slaughterhouses

Detection of L. monocytogenes

A total of 14 L. monocytogenes isolates were detected from 287 swabs collected from poultry and cattle slaughterhouses (4.87%). All isolates were detected in poultry slaughterhouses (A, B, and C), which represented 169 swabs (8.28%). L. monocytogenes was not detected in any of the 118 samples from cattle slaughterhouses (D and E). Detection points for isolates are listed in Table 2. The present study is the first investigation of L. monocytogenes biofilms in poultry and cattle slaughterhouses facilities, equipment, and utensils in the region of the Federal District in Brazil. In this region, reports have described the presence of L. monocytogenes isolated from different sources [48,49](49). The prior findings corroborate the present results. The absence of L. monocytogenes in the cattle slaughterhouses differs from Palma et al. 2016 [49], that reported the presence of L. monocytogenes in samples from cattle slaughterhouses. The difference between the studies may be related to the small number of industries and visits that were made, which was due to the resistance of facilities to participate in the study. The difference may also reflect the adequate hygiene and sanitation conditions of these establishments.


Table 2. Detection of Listeria monocytogenes in swabs from poultry slaughterhouse facilities, equipment and utensils located in the Federal District and State of Goiás, Brazil.
Region and establishment 	DF	DF	DF	DF	DF	GO	GO	DF					
Identification	A	A	A	A	A	C	C	B					
Swab collection points	Collection 1 
No. of swabs=16	Collection 2 
No. of swabs=16	Collection 3 
No. of swabs=16	Collection 4 
No. of swabs=15	Collection 5 
No. of swabs=25	Collection 6
No. of swabs=25	Collection 7
No. of swabs=25	Collection 8
No. of 
swabs=31	Total
Listeria monocytogenes
strains	Total swabs obtained from each collection point	Percentage of positive samples in each collection point (%)	Identification of isolates detected in each collection point 	
Facilities									4	56	7.14		
Floor in clean area 	0	0	0	0	0	0	0	0	0	1	0.0		
Drains in clean area 	0	0	1	0	0	0	1	1	3	38	7.89	63A-1;69A-2; 117A-3	
Drains in dirty area	0	0	0	0	0	0	0	0	0	2	0.0		
Walls in clean area	0	0	0	0	0	0	1	0	1	13	0.0	76A-2	
Walls in dirty area	0	0	0	0	0	0	0	0	0	2	0.0		
Equipment and utensils									10	113	8.84		
Evisceration table	0	0	0	0	0	0	2	0	2	35	5.71	74A-2; 77A-2	
Mats in clean area 	0	0	0	0	0	3	0	0	3	33	9,09	45A-2; 52A-2; 59A-2	
Chutes of meat	0	0	0	0	0	2	3	0	5	13	38.46	42A-2; 54A-2; 72A-2; 78A-2; 88A-2	
Chutes of carcass	0	0	0	0	0	0	0	0	0	14	0.0		
Chutes of bones and viscera	0	0	0	0	0	0	0	0	0	5	0.0		
Hooks	0	0	0	0	0	0	0	0	0	6	0.0		
Machinery	0	0	0	0	0	0	0	0	0	7	0.0		
Total L. monocytogenes isolates obtained in each collection	0	0	1	0	0	5	7	1	14				
Total swabs obtained in each collection	16	16	16	15	25	25	25	31		169			


The detection of L. monocytogenes in poultry slaughterhouses is similar to findings described by other authors in other regions of Brazil [4,50–52]. The detection of L. monocytogenes at the points described in the present study agrees with prior descriptions of the detection on stainless steel tables [4,51], mats [4], chutes [52], and drains [12,28](28). All collections were performed after the environment was cleaned at least once, suggesting the persistence of the microorganism, possibly due to the ability to adhere to surfaces and form biofilms [53–55]. At one collection point, a chute in slaughterhouse C, L. monocytogenes was detected in two samples collected on different days, suggesting the presence of biofilm at the site. In two different studies carried out in the United States, Berrang et al. [28,56] used serial collection from the same points within the slaughter plant at different times to characterize the presence of persistent L. monocytogenes isolates in the environments of poultry slaughterhouses. The authors concluded that the persistence of this microorganism in drains is related to the formation of biofilms in this type of installation [56]. In Brazil, similar to the present study, Sereno et al. 2019 [57] performed serial swab collections of walls, drains, tables, mats, and floors in a swine slaughterhouse in the southern region of the country. The persistence of L. monocytogenes strains was detected at the same collection point over time and was related to the presence of biofilms in that location. Areas of bacterial persistence have included drains [28,56] and a mat [57]. We also detected the presence of L. monocytogenes at these sites, although there was no detection at the same site more than once, except for the chute. This is the first report of biofilm characterization from repeated swab collections at specific sites of facilities and equipment / utensils in Brazilian poultry slaughterhouses during different visits over a period.

Detection of Salmonella spp. 

One Salmonella spp. was detected in 287 (0.34%) swabs collected in the cattle and poultry slaughterhouses. The isolate originated from a drain collected from a dirty area of poultry slaughterhouse B. This is the first investigation of Salmonella spp. in a processing plant of a poultry slaughterhouse located in the Federal District. However, other reports have described of the occurrence of Salmonella spp. in poultry carcasses/viscera produced and/or sold in this region [36]. The low detection in poultry slaughterhouses and absence in cattle slaughterhouses may reflect the efficiency of self-control, disinfection, and sanitation programs in these industries, the reduced number of visits resulting from the resistance of industries to allow internal access for the collection, and / or to the implementation of control programs by the government with the aim of reducing the incidence of Salmonella spp. to protect consumers [58–60].

Serotyping analysis by PCR and evaluation of genetic variability of Listeria monocytogenes by PFGE

All 14 isolates were classified by PCR as 1/2a or 3a serotypes, belonging to lineage II [61–63], with amplification of the lmo0737 gene (691pb). These results corroborate reports in the literature that point to serotype 1/2a, along with serotypes 1/2b and 1/2c, as one of the most commonly detected serotypes in food samples and food production environments of animal origin [64]. The polymorphic results obtained by PFGE identified 11 distinct pulsotypes that were grouped in four clusters (I, II, III, IV) with a similarity threshold of 80% [65] (Fig 1). To analyze strain dissemination within industries and in the studied regions, clonal variations were considered only for those isolates presenting 100% identity with each other [66]. Pulsotype 9 was the most detected (3/14 isolates), followed by pulsotype 5 (2/14 isolates). The same pulsotype was detected at different collection points within the same industry (pulsotypes 5 and 9, slaughterhouse C located in Goiás; Table 2), suggesting the dissemination of these two clones within this establishment. Pulsotype 9 was detected at different sites and in different visits within this slaughterhouse. Thus, although sanitization was performed between sample collections, the fact that the same strain was detected in different collections within the same establishment indicates the presence of biofilms. This finding corroborates the reports of Berrang et al. 2005 [28], Berrang et al. 2010 [56], Camargo et al. 2015 [13], and Sereno et al. 2019 [57]. Pulsotype 9 was also detected in a drain sample obtained in slaughterhouse B located in the Federal District, suggesting regional dissemination of the strain. The foregoing results highlight the importance of using molecular typing methods with high discriminatory power, such as PFGE, to investigate the dissemination and persistence of strains within food processing plants [12,13,67,68]. These typing methods are valuable when WGS analysis cannot be performed. Similar to the results of this study in relation to the use of PFGE, Camargo et al. 2015 [13] described serial collection of samples during different visits from environmental locations, cuts of meat, and the hands of employees working in a cattle slaughterhouse in the state of Minas Gerais. This allowed the identification of cross-contamination between these points and the persistence of specific pulsotypes. The present results confirm the persistence of two pulsotypes (5 and 9) in slaughterhouse C, and possible regional strain dissemination (pulsotype 9). However, due to the low number of isolates identified in slaughterhouses A and B, it was not possible to confirm the persistence of isolates within these establishments. However, the detection of isolates before starting operations in the slaughterhouses (pre-sanitization) and after slaughter (post-sanitization) suggests the continuous presence of this microorganism in preformed biofilms in these facilities.

Figure 1. Dendrogram and PFGE patterns of 14 isolates of Listeria monocytogenes restricted with AscI. The data were analyzed using the BioNumerics software. Discrimination of pulsotypes, clusters, origins of isolates (Federal District, DF; or Goiás, GO), establishment of origin (A, B or C), and isolate identification are presented.

Salmonella spp. serotyping by WGS analysis

WGS analysis of Salmonella spp. isolate identified it as Salmonella enterica serovar Minnesota belonging to ST548 [69]. This result differs from other studies in the investigated regions, which reported a higher occurrence of serovar Enteritidis [36,70,71]. The S. Minnesota ST548 from chicken carcass, chicken feet, and mechanically recovered meat have been reported in Brazil (São Paulo and Minas Gerais, São Paulo, and Federal District, respectively) [72]. On the other hand, this is the first report of its presence in a poultry slaughterhouse located in the Federal District. 

Sequencing analysis of inlA gene of L. monocytogenes isolates

PMSCs were not detected in any of the 14 L. monocytogenes isolates. These results corroborate the results of Medeiros et al. 2020 [73]. The latter author reported the absence of PMSCs in L. monocytogenes isolates from samples collected in slaughterhouse poultry drains in the region of the Federal District. However, the present results differ from those of Manuel et al. 2015 [74] in the United States and Camargo et al. 2019 [75] in Brazil. Both studies reported a higher occurrence of PMSCs in strains isolated from food samples and food production environments. The occurrence of PMSCs in L. monocytogenes isolates is associated with attenuated invasion phenotypes [18,76]. Isolates from human listeriosis have less frequent PMSCs in the inlA gene than isolates from food [74,75,77]. The presently reported absence of PMSCs is an important finding to public health, since the integrity of the inlA gene is necessary to promote the internalization of this pathogen in host cells [18,78].

Cell invasion and adhesion assays

All 14 L. monocytogenes isolates adhered to the Caco-2 cells surface, with an adhesion capacity that varied from 17.38% to 57.14%. Seven of 14 isolates (50%) were able to invade Caco-2 cells, with an invasion capacity varying from 5.16% to 34.27% (Fig 2). The isolates that were able to invade belonged to clusters I and III, and pulsotypes 1 (76A-2), 4 (54A-2), 7 (74A-2), 9 (59A-2, 78A-2 e 117A-3), and 10 (88A-2) (Fig 2). The three isolates belonging to pulsotype 9 were able to invade Caco-2 cells, although with different capacities. The invasion capacity observed in seven of the 14 isolates, in which the presence of PMSCs was not detected, corroborates the findings of Nightingale et al. 2005 [18]. The latter authors reported that isolates without PMSCs invaded Caco-2 cells significantly more than isolates with PMSCs in the inlA gene. However, the results differed in relation to the other seven isolates. Despite not having PMSCs, these isolates were not able to invade Caco-2 cells. Several studies have demonstrated the central role of inlA for cell invasion [18,77,79]. However, other elements may also be important for the internalization of L. monocytogenes in different cell types, such as internalin B (80) and listeriolysin O [81]. Furthermore, differences in invasion capacity among isolates with the complete gene inlA have been described, indicating that other factors may be related to the efficiency of invasion of this pathogen [82].

Figure 2. Results of invasion and cell adhesion tests in Caco-2 cells of 14  Listeria monocytogenes isolates. The values shown are the simple average of the results, and the bars represent the standard deviation.

Antibiograms and detection of resistance genes

L. monocytogenes

Resistance or intermediate sensitivity was detected for seven of the eight antimicrobials tested (Table 3). Six of the 14 isolates (42.9%) were sensitive to all the tested antibiotics. Eight isolates (57.1%) displayed resistance or intermediate sensitivity. The absence of resistance to ampicillin and chloramphenicol corroborates the results observed by other authors in isolates from food sources and food production environments [49,83–85]. Surveillance of the occurrence of ampicillin resistance is important for public health, since this drug is one of the treatments of choice for human listeriosis [86]. The sensitivity to chloramphenicol can be explained by the ban on the use of this antibiotic for the production of food of animal origin in Brazil since 2003 [87]. The present results of resistance and intermediate sensitivity for ciprofloxacin, erythromycin, and gentamicin (42.8%, 35.7%, and 35.7% of L. monocytogenes isolates, respectively) contrast with those observed by other authors, such as Teixeira et al. 2020 [85], who reported the absence of resistance for these three antibiotics in isolates from beef cuts in the state of Mato Grosso, Brazil. The present findings of resistance to gentamicin and erythromycin are a public health concern, since they are the drugs of choice for the treatment of listeriosis in specific cases, such as the use of gentamicin associated with penicillin as an alternative to ampicillin, and the use of erythromycin in the treatment of pregnant women [86,88]. The low occurrence of resistance to doxycycline detected in this study corroborates the findings of Vitas et al. 2007 [89], who reported the low occurrence of resistance in isolates of L. monocytogenes obtained in food and human clinical cases in Spain. The low detection of resistance to tetracycline (7.1% of isolates) corroborates the findings of Haubert et al. 2016 [84] in isolates from food and food production environments in the southern region of Brazil. However, the present results differ from those described by Palma et al. 2016 [49] and Camargo et al. 2015 [83], in which there was no detection of resistance to tetracycline in isolates from samples of beef cuts, cattle slaughterhouse environment, and clinical cases in Brazil. The highest occurrence of antimicrobial resistance detected in this study was in relation to sulfonamides, which was found in eight isolates (57.1%). These findings corroborate the results of other authors, who also reported the pronounced prevalence of resistance to this class of antimicrobial in isolates from food and food production environments [49,85,91]. Sulfonamides associated with trimethoprim are considered as the second-choice treatment for human listeriosis [86]. Therefore, the detection of resistance to this class of antimicrobials may indicate a risk to human health.

Table 3. Antibiogram of 14 Listeria monocytogenes isolates from poultry slaughterhouses located in the region of the Federal District and Goiás, performed by means of disk diffusion tests for antimicrobial resistance (CLSI, 2020).
Antimicrobial
and class	No. of resistant isolates (%)	No. of isolates with an intermediate sensitivity (%)	No. of sensitive isolates (%)	Total intermediate resistance isolates (%)	
Sulfonamides
(sulfonamide)	8 (57.1)	0 (0.0)	6 (42.9)	8 (57.1)	
Erythromycin
(macrolide)	4 (28.6)	1 (7.1)	9 (64.3)	5 (35.7)	
Ciprofloxacin
(quinolone)	3 (21.4)	3 (21.4)	8 (57.2)	6 (42.9)	
Gentamicin
(aminoglycoside)	4 (28.6)	1 (7.1)	9 (64.3)	5 (35.7)	
Chloramphenicol
(chloramphenicol)	0 (0.0)	4 (28.6)	10 (71.4)	4 (28.6)	
Tetracycline
(tetracycline)	1 (7.1)	1 (7.1)	12 (85.8)	2 (14.3)	
Doxycycline
(tetracycline)	1 (7.1)	0 (0.0)	13 (92.9)	1 (7.1)	
Ampicillin
(beta-lactamases)	0 (0.0)	0 (0.0)	14 (100)	0 (0.0)	

Four of 14 antimicrobial resistance genes were detected in the L. monocytogenes isolates. The most detected gene was tetC (11/14 isolates, 78.6%), followed by the tetB gene (5/14 isolates, 35.71%), and the ermB and tetM genes (2/14 isolates, 14.28%). The ermA, ermC, cat1, sulI, aaC (3)-1, cmlA, ereA, SHV, and ampC genes were not detected. The detection of the ermB gene corroborates the findings of Haubert et al. [84], who reported this gene in an isolate of L. monocytogenes from a poultry slaughterhouse environment in the southern region of Brazil. Only two of four isolates of L. monocytogenes that showed erythromycin resistance phenotype in the disk diffusion test (Table 2) harbored one of the erythromycin-related resistance genes investigated. It is possible that the isolates that did not have ermA, ermB or ermC genes rather harbored genes related to other mechanisms of resistance to this antimicrobial, such as msr(A) or mef(A) genes [8]. Alternatively, the resistance of these isolates may be related to a chromosomal mutation [92].
The presence of tetM gene in two isolates (14.28%) corroborate the results of Bertrand et al. [93], who reported the presence of this gene in isolates with phenotype of resistance to tetracycline in strains from human clinical samples and swine and poultry slaughterhouses environments in Belgium and France. Several studies reported the tetM gene as the genotype most commonly related to tetracycline resistance in Listeria isolates [93,94]. These findings differ from the present finding that the most prevalent gene related to tetracycline resistance was tetC. Despite the detection of tetC in 11 of 14 isolates (78.6%), only two isolates displayed a resistance or intermediate sensitivity phenotype to tetracycline in the disk diffusion test (Table 3). One of these two isolates harbored two of the four genes related to the resistance against this drug (tetC and tetM), and was classified as resistant in the antibiogram. The other isolate harbored only the tetC gene and showed intermediate sensitivity to this antimicrobial. The other nine isolates with the tetC gene did not show resistance to tetracycline in the disk diffusion test. Therefore, the unique presence of tetC in the isolates did not confer a resistance phenotype to tetracycline, suggesting that the genes do not necessarily trigger the resistance phenotype, which may be related to the presence of mutations that lead to gene dysfunction [95].
Despite the occurrence of antimicrobial resistance to gentamicin in five of the 14 isolates in the disk diffusion test (Table 3), the presence of the aac (3)-1 gene was not detected in any isolate. It is possible that resistance to gentamicin in these isolates was related to the presence of other genes not investigated in this study, since more than 170 genes related to resistance to aminoglycosides have been described in bacteria [8]. Presently, resistance to sulfonamides was observed in eight of 14 isolates in the disk diffusion test, but no isolates harbored the sulI gene. It is possible that this resistance was associated with other mechanisms, such as the presence of other genes related to sulfonamides resistance, for instance sul2, folP, or thyA genes, with the last two genes previously described in L. monocytogenes [96].
Only intermediate sensitivity to chloramphenicol was detected in four of 14 isolates in the disk diffusion test. The cmlA or cat1 genes were not detected. The intermediate sensitivity observed in this study can be related to cross-resistance due to the presence of other genes that were not investigated, such as floR [97]. It is worth mentioning that in Brazil this antimicrobial has been banned from use in animal production since 2003 [98]. This selection pressure was not a factor. The absence of the ampC and bla_SHV_ genes is consistent with the results of the absence of ampicillin resistance phenotype in the disk diffusion test.
Four of the 14 isolates (28.5%) showed resistance to three or more classes of antimicrobials, and were classified as multi-resistant [99]. All were from establishment C. Two isolates (54A-2 and 88A-2) adhered and invaded Caco-2 cells. The description of L. monocytogenes multi-resistant isolates has increased in the literature [84,94,100]. All multi-resistant isolates detected in this study showed resistance to antimicrobials used in the treatment of human listeriosis (gentamicin and erythromycin) [86]. There is a possibility of the transfer of genes from multidrug-resistant isolates to others. Further studies are needed to elucidate all the resistance mechanisms observed in these isolates and their actual potential as a source of resistance transfer to other microorganisms.

Salmonella enterica Minnesotaspp.

Resistance or intermediate sensitivity of Salmonella Minnesotaspp. isolate was detected for nine of 13 antimicrobials tested (Table 3). The resistance to tetracycline and ampicillin, and the sensitivity to ciprofloxacin and gentamicin were similar to the results obtained by Dantas et al. [101]. The latter authors reported this same pattern of resistance in isolates of Salmonella spp. from the poultry slaughterhouse environment in the state of São Paulo. The detection of resistance to ampicillin, cephalothin, and sulfonamide bases, in addition to sensitivity to ciprofloxacin, corroborate the findings of Cunha-Neto et al. 2018 [102] in isolates of Salmonella spp. from chicken carcasses of a slaughterhouse in the state of Mato Grosso. However, these authors reported the detection of resistance to gentamicin and chloramphenicol, and sensitivity to nalidixic acid, which differs from the present results. The detection of resistance to nalidixic acid in isolates of Salmonella spp. has been widely reported [103–105]. Meta-analyses of publications for a period of 20 years in Brazil have revealed the increased occurrence of resistance to this quinolone in isolates from human samples and chicken meat [106]. Resistance to nalidixic acid may be due to the prolonged and widespread use of this antimicrobial in human and veterinary medicine, since this was the first drug in the quinolone class to have clinical use [107]. Similar to nalidixic acid resistance, the resistance to tetracycline, sulfonamides, and beta-lactams may be related to the overuse of these antimicrobials in the different stages of the chicken meat production chain in Brazil [108].
The present study detected resistance to the three generations of cephalosporins. Cephalosporins, especially those of the third and fourth generation, are important for human and animal health [109]. Resistance to cephalosporins, including third and fourth generation drugs, has been reported in recent years [110,111]. This detection of resistance, especially to ceftazidime, is a public health concern and highlights the need for vigilance and parsimony in the use of antimicrobials in veterinary medicine. In recent years, the Brazilian government has promoted restrictions on the use of antimicrobials in the production of animal foods, such as the ban on the use of chloramphenicol and nitrofurans [98], colistin sulfate [112], and more recently the ban on the use of tylosin, lincomycin, and tiamulin [113].
WGS analysis of Salmonella isolate (GenBank access number JABBEB000000000.1) detected the tetA, sul2, aac (6')-Iaa, bla_CMY-2_, and qnrB19 genes; as well as a mutation in the parC gene (T57S, ACC →AGC, causing the mutation T→S; Table 4). All resistance genes we detected have been described in Salmonella spp. isolates in Brazil [72,114]. The concomitant presence of plasmid-mediated quinolone resistance (PMQR) via the qnrB19 gene, and mutation in the region determining quinolone resistance (QRDR) in the parC gene of Topoisomerase IV were also observed. These genetic characteristics were confirmed with the expression of resistance to nalidixic acid, but there was no detection of resistance to ciprofloxacin in the antibiograms. Despite this, the presence of the qnrB19 gene and other genes related to PMQR may be responsible for the reduced susceptibility to quinolones, which may facilitate the selection of less susceptible isolates and consequent failure in the treatment with this class of drugs [115]. The detection of PMQR in this study highlights the need for surveillance about the persistence of quinolone resistance genes in the poultry production chain. The bla_CMY-2_ gene is the plasmid gene related to the production of beta-lactamases (pAmpC) most commonly detected globally [116]. The presence of this gene corroborates the results of resistance to beta-lactams observed in the disk diffusion test (ampicillin, cephalothin, cefazolin, and ceftazidime). Its presence is another public health concern considering the importance of beta-lactams to the human and animal health, especially third generation cephalosporins [109]. The presence of the aac-(6')-Iaa gene related to resistance to aminoglycosides did not confer resistance to gentamicin, the only base of this class of antimicrobials evaluated in the disk diffusion. This discrepancy between phenotype and genotype was also observed in Salmonella spp. isolates from different samples in the poultry production chain in the states of São Paulo, Bahia, and Minas Gerais [72]. On the other hand, the occurrence of resistance to tetracycline and sulfonamides is related to the presence of the tetA and sul2 genes, respectively. The present absence of colistin resistance genes corroborates Monte et al. [72], who reported the absence of these genes in Salmonella spp. isolates obtained from samples of swine and poultry production chains from different regions of Brazil, including the Federal District.

Table 4. Antibiogram and detection of antimicrobial resistance genes from the Salmonella enterica serovar Minnesota isolate from a poultry slaughterhouse located in the Federal District, carried out using the disk diffusion test (CLSI, 2020) and WGS, respectively.
Antibiogram (tested drugs)	Antibiogram result 	Antimicrobial resistant genes	
Nalidixic acid	R	qnrB19	
Amoxicillin	R	bla_CMY-2_	
Ampicillin	R	bla_CMY-2_	
Cephalothin	R	bla_CMY-2_	
Cefazoline	R	bla_CMY-2_	
Ceftazidime	R	bla_CMY-2_	
Ciprofloxacin	S	-	
Chloramphenicol	S	-	
Colistin	S	-	
Doxycycline	I	-	
Gentamicin	S	aac(6')-1aa	
Sulfonamides	R	sul2	
Tetracycline	R	tetA	
S, sensitive; I, intermediate; R, resistant.

The resistance profile observed in this Salmonella spp. isolate classified it as a multi-resistant isolate [99]. This characteristic, associated with the presence of mobile genetic elements related to resistance, such as the presence of PMQR and pAmpC, suggests a potential public health risk. In addition, the occurrence of resistance to quinolones and cephalosporins, associated with the presence of genetic elements related to this phenotype, are of special concern, given the importance of these drugs for human health [109]. 

In vitro biofilm formation capacity

L. monocytogenes

During incubation at 37°C for 24 h, 11 of 14 isolates (78.57%) were capable of biofilm formation and were classified as weak biofilm formers. During incubation at 12°C for 168 h, nine of 14 isolates (64.3%) were capable of biofilm formation. All nine were weak biofilm formers (Fig 3). The biofilm formation capacity by L. monocytogenes isolates corroborated other studies reporting their ability to adhere and form biofilms on abiotic surfaces [117–119]. The observed results of poor biofilm formation capacity of most isolates (78.57%) at 37°C corroborated the findings of Harvey et al. 2007 [117]. The latter authors reported that 90% of the tested L. monocytogenes isolates were weak biofilm forming bacteria. There was no significant difference (p=0.2450) in the biofilm formation capacity of the isolates when incubated at 37°C for 24 h and 12°C for 168 h. The finding that nine of the 14 isolates could form biofilms during incubation at 12°C highlights the risks that these isolates can pose to cattle and poultry slaughter facilities in the event of failures in the hygiene and sanitation of the environments.

Figure 3. Results of the in vitro biofilm formation capacity test in polystyrene microplates (Djordjevic et al., 2002) of 12 Listeria monocytogenes isolates with biofilm formation capacity at 37°C and / or 12°C. The bars represent the average reading of the optical density of each test for each isolate (ODi), all performed in triplicate, subtracted from the average of the optical density of the negative control for each repetition (ODn).

Most isolates in this study demonstrated the ability to form biofilms in at least one of the conditions tested. These findings, allied with the findings of repeated detection of clonal variations within the same industry at different points and different visits, in addition to the identification of the same pulsotype in another slaughterhouse in another region, supports the possibility of L. monocytogenes biofilms in poultry slaughterhouses located in the Federal District and Goiás. The detection of the biofilm formation capacity of the isolates, especially at the 12°C temperature observed inside slaughterhouses, indicates the possibility that these isolates can adhere to surfaces, including those of equipment and utensils, which suggests the possibility of biofilm formation in the event of failures in the hygiene procedures. This creates a potential public health risk due to potential food cross-contamination.

Salmonella spp.

Salmonella spp. isolate was capable of biofilm formation, albeit weakly, in both conditions tested. These results corroborate those of Yin et al. 2018 [120]. The authors reported the capacity of biofilm formation of Salmonella spp. isolates from beef production environments in China at different temperatures, including 12°C and 37°C. The present results suggest the potential risk to public health by the Salmonella spp. isolate in the slaughterhouse environment, since, in addition to its multi-resistance characteristic, this isolate may adhere to abiotic surfaces, being a potential contaminant of processed foods.

Conclusions

	The presence of L. monocytogenes and Salmonella spp. was identified for the first time in poultry slaughterhouses located in the Federal District and in the State of Goiás, Brazil. The bacteria were detected on equipment and utensils, especially chutes, which were the main routes of contamination by L. monocytogenes within the evaluated plants. There was no detection in cattle slaughterhouses. The consistent and repeated collection of the points allowed the characterization of biofilms in the environments of these industries, which was confirmed in 12 of L. monocytogenes and in Salmonella spp. isolates by the in vitro biofilm formation capacity test performed at 12°C and/or 37°C. The PFGE patterns allowed to evaluate the dissemination of specific pulsotypes both within an industry and in the studied regions. Sequencing of the inlA gene from L. monocytogenes isolates demonstrated the absence of PMSCs in all isolates. These results, along with the adhesion and cell invasion results, suggest their virulence potential. The detection of resistance to antimicrobials of public health importance in L. monocytogenes and Salmonella spp. isolates, in addition to the detection of multidrug-resistant isolates, also underlines the potential risk for the consumer due to the possibility of food cross-contamination via contact with biofilms formed on abiotic surfaces. The results of this study indicate the importance of research that supports surveillance in slaughterhouse environments for possible adjustments in environmental hygiene and sanitation procedures. In addition, the detection of isolates with pathogenic potential related to the capacity for cell invasion, the ability to form biofilms, and with antimicrobial resistance, indicate the need to assess the risk for the population as an instrument of public health protection.

Nucleotide sequence accession number
This Whole Genome Shotgun project has been deposited at DDBJ/ENA/GenBank under the accession JABBEB000000000 (SRA: SRR12606957). The version described in this paper is version JABBEB000000000.1.

Acknowledgements
We thank the staff from the Laboratory of Veterinary Microbiology and Laboratory of Genic Therapy for providing help when needed.

Conflict of Interest
The authors declare that they have no conflict of interest.

References

1. 	Møretrø T, Langsrud S. Residential Bacteria on Surfaces in the Food Industry and Their Implications for Food Safety and Quality. Compr Rev Food Sci Food Saf. 2017 Sep 1;16(5):1022–41. 
2. 	Ferreira V, Wiedmann M, Teixeira P, Stasiewicz MJ. Listeria monocytogenes persistence in food-associated environments: Epidemiology, strain characteristics, and implications for public health. Vol. 77, Journal of Food Protection. J Food Prot; 2014. p. 150–70. 
3. 	Carrascosa C, Raheem D, Ramos F, Saraiva A, Raposo A. Microbial biofilms in the food industry—a comprehensive review. Int J Environ Res Public Health. 2021 Feb 2;18(4):1–31. 
4. 	Rodrigues L, Dos Santos L, Tagliari V, Rizzo N, Trenhago G, de Oliveira A, et al. Quantification of biofilm production on polystyrene by Listeria, Escherichia coli and Staphylococcus aureus isolated from a poultry slaughterhouse. Braz J Microbiol. 2010;41(4). 
5. 	Larsen MH, Dalmasso M, Ingmer H, Langsrud S, Malakauskas M, Mader A, et al. Persistence of foodborne pathogens and their control in primary and secondary food production chains. Food Control. 2014 Oct 1;44:92–109. 
6. 	Kasnowski MC, Mantilla SPS, Oliveira LAT, Franco RM. Formação de biofilme na indústria de alimentos e métodos de validação de superfícies. Rev Científica Eletrônica da Med Veterinária. 2010;VIII(15). 
7. 	FAO F and AO of the UN, WHO WHO. Principles and Guidelines for the Conduct of Microbiological Risk Assessment. FAO Agric Consum Prot. 2007;63:68. 
8. 	Luque-Sastre L, Arroyo C, Fox EM, McMahon BJ, Bai L, Li F, et al. Antimicrobial Resistance in Listeria Species. In: Antimicrobial Resistance in Bacteria from Livestock and Companion Animals. Washington, DC, USA: ASM Press; 2018. p. 237–59. 
9. 	Carpentier B, Cerf O. Review - Persistence of Listeria monocytogenes in food industry equipment and premises. Vol. 145, International Journal of Food Microbiology. Elsevier; 2011. p. 1–8. 
10. 	Datta AR, Burall LS. Serotype to genotype: The changing landscape of listeriosis outbreak investigations. Vol. 75, Food Microbiology. Academic Press; 2018. p. 18–27. 
11. 	Doumith M, Buchrieser C, Glaser P, Jacquet C, Martin P. Differentiation of the major Listeria monocytogenes serovars by multiplex PCR. J Clin Microbiol. 2004 Aug;42(8):3819–22. 
12. 	Berrang ME, Meinersmann RJ, Northcutt JK, Smith DP. Molecular characterization of Listeria monocytogenes isolated from a poultry further processing facility and from fully cooked product. J Food Prot. 2002 Oct 1;65(10):1574–9. 
13. 	Camargo AC, Dias MR, Cossi MVC, Lanna FGPA, Cavicchioli VQ, Vallim DC, et al. Serotypes and pulsotypes diversity of Listeria monocytogenes in a beef-processing environment. Foodborne Pathog Dis. 2015 Apr 1;12(4):323–6. 
14. 	Maury MM, Tsai YH, Charlier C, Touchon M, Chenal-Francisque V, Leclercq A, et al. Uncovering Listeria monocytogenes hypervirulence by harnessing its biodiversity. Nat Genet. 2016 Mar 1;48(3):308–13. 
15. 	Lecuit M, Vandormael-Pournin S, Lefort J, Huerre M, Gounon P, Dupuy C, et al. A transgenic model for listeriosis: Role of internalin in crossing the intestinal barrier. Science. 2001 Jun 1;292(5522):1722–5. 
16. 	Orsi RH, Ripoll DR, Yeung M, Nightingale KK, Wiedmann M. Recombination and positive selection contribute to evolution of Listeria monocytogenes inlA. Microbiology. 2007 Aug;153(8):2666–78. 
17. 	Poyart C, Trieu-Cuot P, Berche P. The inlA gene required for cell invasion is conserved and specific to Listeria monocytogenes. Microbiology. 1996;142(1):173–80. 
18. 	Nightingale KK, Windham K, Martin KE, Yeung M, Wiedmann M. Select Listeria monocytogenes subtypes commonly found in foods carry distinct nonsense mutations in inlA, leading to expression of truncated and secreted internalin A, and are associated with a reduced invasion phenotype for human intestinal epithelial ce. Appl Environ Microbiol. 2005 Dec;71(12):8764–72. 
19. 	Ferrari RG, Rosario DKA, Cunha-Neto A, Mano SB, Figueiredo EES, Conte-Juniora CA. Worldwide epidemiology of Salmonella serovars in animal-based foods: A meta-analysis. Appl Environ Microbiol. 2019 Jul 1;85(14). 
20. 	Joseph B, Otta SK, Karunasagar I, Karunasagar I. Biofilm formation by Salmonella spp. On food contact surfaces and their sensitivity to sanitizers. Int J Food Microbiol. 2001 Mar 20;64(3):367–72. 
21. 	Leriche V, Carpentier B. Limitation of adhesion and growth of Listeria monocytogenes on stainless steel surfaces by Staphylococcus sciuri biofilms. J Appl Microbiol. 2000;88(4):594–605. 
22. 	Sinde E, Carballo J. Attachment of Salmonella spp. and Listeria monocytogenes to stainless steel, rubber and polytetrafluorehtylene: The influence of free energy and the effect of commercial sanitizers. Food Microbiol. 2000 Aug 1;17(4):439–47. 
23. 	McEwen SA, Collignon PJ. Antimicrobial Resistance: a One Health Perspective. In: Antimicrobial Resistance in Bacteria from Livestock and Companion Animals. American Society of Microbiology; 2018. p. 521–47. 
24. 	World Health Organization. Global Antimicrobial Resistance Surveillance System (GLASS) Report Early implementation 2017-2018. 2018. 
25. 	World Health Organization. Global Action Plan on Antimicrobial Resistance. 2015. 
26. 	Nicolau AI, Bolocan AS. Sampling the processing environment for listeria. In: Methods in Molecular Biology. Humana Press Inc.; 2014. p. 3–14. 
27. 	Barros MAF, Nero LA, Silva LC, D'Ovidio L, Monteiro FA, Tamanini R, et al. Listeria monocytogenes: Occurrence in beef and identification of the main contamination points in processing plants. Meat Sci. 2007 Aug;76(4):591–6. 
28. 	Berrang ME, Meinersmann RJ, Frank JF, Smith DP, Genzlinger LL. Distribution of Listeria monocytogenes subtypes within a poultry further processing plant. J Food Prot. 2005;68(5):980–5. 
29. 	Pan Y, Breidt F, Kathariou S. Resistance of Listeria monocytogenes biofilms to sanitizing agents in a simulated food processing environment. Appl Environ Microbiol. 2006 Dec;72(12):7711–7. 
30. 	Vestby LK, Møretrø T, Langsrud S, Heir E, Nesse LL. Biofilm forming abilities of Salmonella are correlated with persistence in fish meal- and feed factories. BMC Vet Res. 2009 May 27;5. 
31. 	Ryser ET, Donnelly CW. 35. Listeria. In: Salfinger Y, Tortorello M Lou, editors. Compendium of Methods for the Microbiological Examination of Foods. 5th ed. American Public Health Association; 2015. 
32. 	D'Agostino M, Wagner M, Vazquez-Boland JA, Kuchta T, Karpiskova R, Hoorfar J, et al. A validated PCR-based method to detect Listeria monocytogenes using raw milk as a food model - Towards an international standard. J Food Prot. 2004;67(8):1646–55. 
33. 	Kérouanton A, Marault M, Petit L, Grout J, Dao TT, Brisabois A. Evaluation of a multiplex PCR assay as an alternative method for Listeria monocytogenes serotyping. J Microbiol Methods. 2010 Feb;80(2):134–7. 
34. 	ISO - ISO 6579:2002 - Microbiology of food and animal feeding stuffs — Horizontal method for the detection of Salmonella spp. 2002. p. 27. 
35. 	Alvarez J, Sota M, Vivanco AB, Perales I, Cisterna R, Rementeria A, et al. Development of a Multiplex PCR Technique for Detection and Epidemiological Typing of Salmonella in Human Clinical Samples. J Clin Microbiol. 2004; 
36. 	de Freitas CG, Santana ÂP, da Silva PHC, Gonçalves VSP, Barros M de AF, Torres FAG, et al. PCR multiplex for detection of Salmonella Enteritidis, Typhi and Typhimurium and occurrence in poultry meat. Int J Food Microbiol. 2010 Apr;139(1–2):15–22. 
37. 	Wick RR, Judd LM, Gorrie CL, Holt KE. Unicycler: Resolving bacterial genome assemblies from short and long sequencing reads. PLoS Comput Biol. 2017 Jun 1;13(6). 
38. 	Zhang S, den Bakker HC, Li S, Chen J, Dinsmore BA, Lane C, et al. SeqSero2: Rapid and improved salmonella serotype determination using whole-genome sequencing data. Appl Environ Microbiol. 2019 Dec 1;85(23). 
39. 	CDC (Centers for Disease Control and Prevention). Standard Operating Procedure for PulseNet PFGE of Listeria monocytogenes. 2017. 
40. 	Gaillard JL, Berche P, Mounier J, Richard S, Sansonetti P. In vitro model of penetration and intracellular growth of Listeria monocytogenes in the human enterocyte-like cell line Caco-2. Infect Immun. 1987;55(11):2822–9. 
41. 	Moroni O, Kheadr E, Boutin Y, Lacroix C, Fliss I. Inactivation of adhesion and invasion of food-borne Listeria monocytogenes by bacteriocin-producing Bifidobacterium strains of human origin. Appl Environ Microbiol. 2006 Nov;72(11):6894–901. 
42. 	Clinical and Laboratory Standards Institute. CLSI M100 ED30:2020. 2020. 
43. 	Chen BY, Pyla R, Kim TJ, Silva JL, Jung YS. Antibiotic resistance in Listeria species isolated from catfish fillets and processing environment. Lett Appl Microbiol. 2010 Jun;50(6):626–32. 
44. 	The European Committee on Antimicrobial Susceptibility Testing. Breakpoint tables for interpretation of MICs and zone diameters. Version 9.0, 2019. 2019. 
45. 	Djordjevic D, Wiedmann M, McLandsborough LA. Microtiter plate assay for assessment of Listeria monocytogenes biofilm formation. Appl Environ Microbiol. 2002;68(6):2950–8. 
46. 	Borges KA, Furian TQ, Souza SN, Menezes R, Tondo EC, Salle CTP, et al. Biofilm formation capacity of Salmonella serotypes at different temperature conditions. Pesqui Vet Bras. 2018 Jan 1;38(1):71–6. 
47. 	Stepanoviæ S, Vukoviæ D, Dakiæ I, Saviæ B, Švabiæ-Vlahoviæ M. A modified microtiter-plate test for quantification of staphylococcal biofilm formation. J Microbiol Methods. 2000;40(2):175–9. 
48. 	Cesar APR, Mesquita AJ de, Prado CS, Nunes IA, Almeida Filho ES de. Listeria spp. e Listeria monocytogenes na produção de salsichas tipo hot dog. Ciência Anim Bras. 2011 Jun 27;12(2):339–52. 
49. 	Palma JM, Lisboa RC, Rodrigues DP, Santos AFM, Hofer E, Santana AP. Caracterização molecular de Listeria monocytogenes oriundas de cortes cárneos bovinos e de abatedouros frigoríficos de bovinos localizados no Distrito Federal, Brasil. Pesqui Vet Bras. 2016;36(10):957–64. 
50. 	Chiarini E, Tyler K, Farber JM, Pagotto F, Destro MT. Listeria monocytogenes in two different poultry facilities: Manual and automatic evisceration. Poult Sci. 2009 Apr 1;88(4):791–7. 
51. 	Loura CAC, Almeida RCC, Almeida PF. The incidente and level of Listeria spp. and Listeria monocytogenes contamination in processed poultry at a poultry processing plant. J Food Saf. 2005 Feb 1;25(1):19–29. 
52. 	Schäfer DF, Steffens J, Barbosa J, Zeni J, Paroul N, Valduga E, et al. Monitoring of contamination sources of Listeria monocytogenes in a poultry slaughterhouse. LWT. 2017 Dec 1;86:393–8. 
53. 	Borucki MK, Peppin JD, White D, Loge F, Call DR. Variation in Biofilm Formation among Strains of Listeria monocytogenes. Appl Environ Microbiol. 2003 Dec 1;69(12):7336–42. 
54. 	Lunden JM, Miettinen MK, Autio TJ, Korkeala HJ. Persistent Listeria monocytogenes strains show enhanced adherence to food contact surface after short contact times. J Food Prot. 2000;63(9):1204–7. 
55. 	Møretrø T, Langsrud S. Listeria monocytogenes : biofilm formation and persistence in food-processing environments. Biofilms. 2004 Apr;1(2):107–21. 
56. 	Berrang ME, Meinersmann RJ, Frank JF, Ladely SR. Colonization of a newly constructed commercial chicken further processing plant with Listeria monocytogenes. J Food Prot. 2010;73(2):286–91. 
57. 	Sereno MJ, Viana C, Pegoraro K, da Silva DAL, Yamatogi RS, Nero LA, et al. Distribution, adhesion, virulence and antibiotic resistance of persistent Listeria monocytogenes in a pig slaughterhouse in Brazil. Food Microbiol. 2019 Dec 1;84:103234. 
58. 	Ministério da Agricultura Pecuária e Abastecimento, Secretaria de Defesa Agropecuária. Instrução Normativa N^o^ 70 , de de 2003 - DispoÞe sobre o Programa de redução de Patógenos, monitoramento microbiológico e controle de Salmonella sp. em carcaças de frangos e perus. 2003. 
59. 	Agência Nacional de Vigilância Sanitária. Programa Nacional de Monitoramento da Prevalência e da Resistência Bacteriana em Frango-PREBAF. 2004. 
60. 	Ministério da Agricultura Pecuária e Abastecimento, Secretaria de Defesa Agropecuária. INSTRUÇÃO NORMATIVA N^o^ 20, DE 21 DE OUTUBRO DE 2016. 2016. 
61. 	Orsi RH, Bakker HC de., Wiedmann M. Listeria monocytogenes lineages: Genomics, evolution, ecology, and phenotypic characteristics. International Journal of Medical Microbiology. 2011 Feb;301(2):79–96. 
62. 	Piffaretti JC, Kressebuch H, Aeschbacher M, Bille J, Bannerman E, Musser JM, et al. Genetic characterization of clones of the bacterium Listeria monocytogenes causing epidemic disease. Proc Natl Acad Sci U S A. 1989;86(10):3818–22. 
63. 	Ward TJ, Ducey TF, Usgaard T, Dunn KA, Bielawski JP. Multilocus genotyping assays for single nucleotide polymorphism-based subtyping of Listeria monocytogenes isolates. Appl Environ Microbiol. 2008 Dec;74(24):7629–42. 
64. 	Swaminathan B, Gerner-Smidt P. The epidemiology of human listeriosis. Vol. 9, Microbes and Infection. Microbes Infect; 2007. p. 1236–43. 
65. 	Tenover FC, Arbeit RD, Goering R V., Mickelsen PA, Murray BE, Persing DH, et al. Interpreting chromosomal DNA restriction patterns produced by pulsed- field gel electrophoresis: Criteria for bacterial strain typing. J Clin Microbiol. 1995;33(9):2233–9. 
66. 	van Belkum A, Tassios PT, Dijkshoorn L, Haeggman S, Cookson B, Fry NK, et al. Guidelines for the validation and application of typing methods for use in bacterial epidemiology. Clin Microbiol Infect. 2007;13(SUPPL. 3):1–46. 
67. 	Wiedmann M. Molecular Subtyping Methods for Listeria monocytogenes. J AOAC Int. 2002 Mar 1;85(2):524–32. 
68. 	Magalhães VD, Ferreira JC, Barelli C, Darini ALC. Eletroforese em campo pulsante em bacteriologia - uma revisão técnica. Rev Inst Adolfo Lutz. 2005;64(2):155–61. 
69. 	Larsen M V., Cosentino S, Rasmussen S, Friis C, Hasman H, Marvig RL, et al. Multilocus sequence typing of total-genome-sequenced bacteria. J Clin Microbiol. 2012 Apr;50(4):1355–61. 
70. 	Moraes DMC, Andrade MA, Minafra-Rezende CS, Barnabé AC de S, Jayme V de S, Nunes IA, et al. Fontes de infecção e perfil de suscetibilidade aos antimicrobianos de Salmonella sp. isoladas no fluxo de produção de frangos de corte. Arq Inst Biol (Sao Paulo). 2014 Sep;81(3):195–201. 
71. 	Moreira G do N, e Rezende CSM, Carvalho RN, de Mesquita SQP, de Oliveira AN, Arruda MLT. Occurrence of Salmonella sp. in carcasses of broilers slaughtered and marketed in cities of state of Goiás, Brazil. Rev Inst Adolfo Lutz. 2008;126–30. 
72. 	Monte DF, Lincopan N, Berman H, Cerdeira L, Keelara S, Thakur S, et al. Genomic Features of High-Priority Salmonella enterica Serovars Circulating in the Food Production Chain, Brazil, 2000–2016. Sci Rep. 2019 Dec 1;9(1):1–12. 
73. 	Medeiros M, Castro VHL de, Mota ALA de A, Pereira MG, De Martinis ECP, Perecmanis S, et al. Assessment of Internalin A Gene Sequences and Cell Adhesion and Invasion Capacity of Listeria monocytogenes Strains Isolated from Foods of Animal and Related Origins. Foodborne Pathog Dis. 2020 Dec 18;18(4):243–52. 
74. 	Manuel CS, Stelten A Van, Wiedmann M, Nightingale KK, Orsi RH. Prevalence and distribution of Listeria monocytogenes inlA alleles prone to phase variation and inlA alleles with premature stop codon mutations among human, food, animal, and environmental isolates. Appl Environ Microbiol. 2015;81(24):8339–45. 
75. 	Camargo AC, Moura A, Avillan J, Herman N, McFarland AP, Sreevatsan S, et al. Whole-genome sequencing reveals Listeria monocytogenes diversity and allows identification of long-term persistent strains in Brazil. Environ Microbiol. 2019 Dec 1;21(12):4478–87. 
76. 	Nightingale KK, Ivy RA, Ho AJ, Fortes ED, Njaa BL, Peters RM, et al. inlA premature stop codons are common among Listeria monocytogenes isolates from foods and yield virulence-attenuated strains that confer protection against fully virulent strains. Appl Environ Microbiol. 2008 Nov;74(21):6570–83. 
77. 	Ferreira da Silva M, Ferreira V, Magalhães R, Almeida G, Alves A, Teixeira P. Detection of premature stop codons leading to truncated internalin A among food and clinical strains of Listeria monocytogenes. Food Microbiol. 2017 May 1;63:6–11. 
78. 	Lecuit M, Ohayon H, Braun L, Mengaud J, Cossart P. Internalin of Listeria monocytogenes with an intact leucine-rich repeat region is sufficient to promote internalization. Infect Immun. 1997;65(12):5309–19. 
79. 	Olier M, Garmyn D, Rousseaux S, Lemaître JP, Piveteau P, Guzzo J. Truncated internalin A and asymptomatic Listeria monocytogenes carriage: In vivo investigation by allelic exchange. Infect Immun. 2005 Jan;73(1):644–8.
80. 	Quereda JJ, Rodríguez-Gómez IM, Meza-Torres J, Gómez-Laguna J, Nahori MA, Dussurget O, et al. Reassessing the role of internalin B in Listeria monocytogenes virulence using the epidemic strain F2365. Clin Microbiol Infect. 2019 Feb 1;25(2):252.e1-252.e4. 
81. 	Phelps CC, Vadia S, Arnett E, Tan Y, Zhang X, Pathak-Sharma S, et al. Relative roles of Listeriolysin O, InlA, and InlB in Listeria monocytogenes uptake by host cells. Infect Immun. 2018 Oct 1;86(10). 
82. 	Ward TJ, Evans P, Wiedmann M, Usgaard T, Roof SE, Stroika SG, et al. Molecular and phenotypic characterization of listeria monocytogenes from U.S. department of agriculture food safety and inspection service surveillance of ready-to-eat foods and processing facilities. J Food Prot. 2010;73(5):861–9. 
83. 	Camargo AC, De Castilho NPA, Da Silva DAL, Vallim DC, Hofer E, Nero LA. Antibiotic resistance of listeria monocytogenes isolated from meat-processing environments, beef products, and clinical cases in Brazil. Microb Drug Resist. 2015 Aug 1;21(4):458–62.
84. 	Haubert L, Mendonça M, Lopes G V., de Itapema Cardoso MR, da Silva WP. Listeria monocytogenes isolates from food and food environment harbouring tetM and ermB resistance genes. Lett Appl Microbiol. 2016 Jan 1;62(1):23–9. 
85. 	Teixeira LAC, Carvalho FT, Vallim DC, Pereira RCL, Neto AC, Vieira BS, et al. Listeria monocytogenes in export-approved beef from mato grosso, brazil: Prevalence, molecular characterization and resistance to antibiotics and disinfectants. Microorganisms. 2020 Jan 1;8(1). 
86. 	Charpentier E, Courvalin P. Antibiotic resistance in Listeria spp. Vol. 43, Antimicrobial Agents and Chemotherapy. American Society for Microbiology; 1999. p. 2103–8. 
87. 	Ministério da Agricultura Pecuária e Abastecimento. Instrução Normativa n^o^ 9 de 27 de junho de 2003. 2003. 
88. 	Craig AM, Dotters-Katz S, Kuller JA, Thompson JL. Listeriosis in Pregnancy: A Review. Obstet Gynecol Surv. 2019 Jun 1;74(6):362–8. 
89. 	Vitas AI, Sánchez RM, Aguado V, García-Jalón I. Antimicrobial susceptibility of Listeria monocytogenes isolated from food and clinical cases in Navarra, Spain. J Food Prot. 2007;70(10):2402–6. 
90. 	Zhang Y, Yeh E, Hall G, Cripe J, Bhagwat AA, Meng J. Characterization of Listeria monocytogenes isolated from retail foods. Int J Food Microbiol. 2007 Jan 1;113(1):47–53. 
91. 	Morvan A, Moubareck C, Leclercq A, Hervé-Bazin M, Bremont S, Lecuit M, et al. Antimicrobial resistance of Listeria monocytogenes strains isolated from humans in France. Antimicrob Agents Chemother. 2010 Jun;54(6):2728–31. 
92. 	Bertrand S, Huys G, Yde M, D'Haene K, Tardy F, Vrints M, et al. Detection and characterization of tet(M) in tetracycline-resistant Listeria strains from human and food-processing origins in Belgium and France. J Med Microbiol. 2005 Dec;54(12):1151–6. 
93. 	Escolar C, Gómez D, Del Carmen Rota García M, Conchello P, Herrera A. Antimicrobial resistance profiles of listeria monocytogenes and listeria innocua isolated from ready-to-eat products of animal origin in Spain. Foodborne Pathog Dis. 2017 Jun 1;14(6):357–63. 
94. 	Davis MA, Besser TE, Orfe LH, Baker KNK, Lanier AS, Broschat SL, et al. Genotypic-phenotypic discrepancies between antibiotic resistance characteristics of Escherichia coli isolates from calves in management settings with high and low antibiotic use. Appl Environ Microbiol. 2011 May;77(10):3293–9. 
95. 	Wang Y, Li X, Osmundson T, Shi L, Yan H. Comparative Genomic Analysis of a Multidrug-Resistant Listeria monocytogenes ST477 Isolate. Foodborne Pathog Dis. 2019 Sep 1;16(9):604–15. 
96. 	Srinivasan V, Nam HM, Nguyen LT, Tamilselvam B, Murinda SE, Oliver SP. Prevalence of antimicrobial resistance genes in Listeria monocytogenes isolated from dairy farms. Foodborne Pathog Dis. 2005 Sep;2(3):201–11. 
97. 	Ministério da Agricultura Pecuária e Abastecimento. Instrução Normativa n^o^ 9 de 27 de junho de 2003. 2003. 
98. 	European Centre for Disease Prevention and Control. The European Union Summary Report on antimicrobial resistance in zoonotic and indicator bacteria from humans, animals and food in 2011. Vol. 11, EFSA Journal. Wiley-Blackwell Publishing Ltd; 2013 May. 
99. 	Maung AT, Mohammadi TN, Nakashima S, Liu P, Masuda Y, Honjoh K ichi, et al. Antimicrobial resistance profiles of Listeria monocytogenes isolated from chicken meat in Fukuoka, Japan. Int J Food Microbiol. 2019 Sep 2;304:49–57. 
100. 	Dantas STA, Camargo CH, Tiba-Casas MR, Vivian RC, Pinto JPAN, Pantoja JCF, et al. Environmental persistence and virulence of Salmonella spp. Isolated from a poultry slaughterhouse. Food Res Int. 2020 Mar 1;129:108835. 
101. 	Da Cunha-Neto A, Carvalho LA, Carvalho RCT, Dos Prazeres Rodrigues D, Mano SB, De Souza Figueiredo EE, et al. Salmonella isolated from chicken carcasses from a slaughterhouse in the state of Mato Grosso, Brazil: Antibiotic resistance profile, serotyping, and characterization by repetitive sequence-based PCR system. Poult Sci. 2018 Apr 1;97(4):1373–81. 
102. 	Vinueza-Burgos C, Baquero M, Medina J, De Zutter L. Occurrence, genotypes and antimicrobial susceptibility of Salmonella collected from the broiler production chain within an integrated poultry company. Int J Food Microbiol. 2019 Jun 16;299:1–7. 
103. 	Yamatogi RS, Oliveira HC, Camargo CH, Fernandes SA, Hernandes RT, Pinto JP, et al. Clonal relatedness and resistance patterns of salmonella corvallis from poultry carcasses in a Brazilian slaughterhouse. J Infect Dev Ctries. 2015 Oct 1;9(10):1161–5. 
104. 	Zhu Y, Lai H, Zou L, Yin S, Wang C, Han X, et al. Antimicrobial resistance and resistance genes in Salmonella strains isolated from broiler chickens along the slaughtering process in China. Int J Food Microbiol. 2017 Oct 16;259:43–51. 
105. 	Voss-Rech D, Potter L, Vaz CSL, Pereira DIB, Sangioni LA, Vargas ÁC, et al. Antimicrobial resistance in nontyphoidal salmonella isolated from human and poultry-related samples in Brazil: 20-year meta-analysis. Foodborne Pathog Dis. 2017 Feb 1;14(2):116–24. 
106. 	Robicsek A, Jacoby GA, Hooper DC. The worldwide emergence of plasmid-mediated quinolone resistance. Lancet Infect Dis. 2006 Oct;6(10):629–40. 
107. 	PAMvet-PR. Levantamento do Uso e Comercialização de Medicamentos Veterinários em Frango de Corte. 2005. 
108. 	World Health Organization. World Health Organization Model List of Essential Medicines, 21st List. 2019. 
109. 	Jeon HY, Seo KW, Kim Y Bin, Kim DK, Kim SW, Lee YJ. Characteristics of third-generation cephalosporin-resistant Salmonella from retail chicken meat produced by integrated broiler operations. Poult Sci. 2019;98(4):1766–74. 
110. 	Ma Y, Li M, Xu X, Fu Y, Xiong Z, Zhang L, et al. High-levels of resistance to quinolone and cephalosporin antibiotics in MDR-ACSSuT Salmonella enterica serovar Enteritidis mainly isolated from patients and foods in Shanghai, China. Int J Food Microbiol. 2018 Dec 2;286:190–6. 
111. 	Ministério da Agricultura Pecuária e Abastecimento. INSTRUÇÃO NORMATIVA N^o^ 45, DE 22 DE NOVEMBRO DE 2016. 2016. 
112. 	Ministério da Agricutura Pecuária e Abastecimento. INSTRUÇÃO NORMATIVA N^o^ 1, DE 13 DE JANEIRO DE 2020. 2020. 
113. 	Vilela FP, Gomes CN, Passaglia J, Rodrigues DP, Costa RG, Casas MRT, et al. Genotypic resistance to quinolone and tetracycline in Salmonella Dublin strains isolated from humans and animals in Brazil. Microb Drug Resist. 2019 Mar 1;25(2):143–51. 
114. 	Jacoby GA, Strahilevitz J, Hooper DC. Plasmid-Mediated Quinolone Resistance. Microbiol Spectr. 2014 Oct 3;2(5). 
115. 	Jacoby GA. AmpC Â-Lactamases. Clin Microbiol Rev. 2009 Jan 1;22(1):161–82. 
116. 	Harvey J, Keenan KP, Gilmour A. Assessing biofilm formation by Listeria monocytogenes strains. Food Microbiol. 2007 Jun;24(4):380–92. 
117. 	Di Bonaventura G, Piccolomini R, Paludi D, D'Orio V, Vergara A, Conter M, et al. Influence of temperature on biofilm formation by Listeria monocytogenes on various food-contact surfaces: Relationship with motility and cell surface hydrophobicity. J Appl Microbiol. 2008 Jun;104(6):1552–61. 
118. 	Magalhães R, Ferreira V, Biscottini G, Brandão TRS, Almeida G, Teixeira P. Biofilm formation by persistent and non-persistent Listeria monocytogenes strains on abiotic surfaces. Acta Aliment. 2017 Mar 1;46(1):43–50. 
119. 	Yin B, Zhu L, Zhang Y, Dong P, Mao Y, Liang R, et al. The Characterization of Biofilm Formation and Detection of Biofilm-Related Genes in Salmonella Isolated from Beef Processing Plants. Foodborne Pathog Dis. 2018 Oct 1;15(10):660–7.
