# Supplementary material for: Molecular characterization of Salmonella spp. and Listeria monocytogenes strains from biofilms in cattle and poultry slaughterhouses located in the federal District and State of Goiás, Brazil
Source: PLoS One. 2021 Nov 12;16(11):e0259687. doi: 10.1371/journal.pone.0259687 (PMC8589217; doi:10.1371/journal.pone.0259687)
Supplement: Attachment — Submitted filename: PONE-D-21-17398_review.docx [file pone.0259687.s003.docx]

PONE-D-21-17398

Molecular characterization of *Salmonella* spp. and *Listeria monocytogenes* strains from biofilms in cattle and poultry slaughterhouses located in the Federal District and State of Goiás, Brazil.

This study aimed to detect biofilm-induced contamination points and the presence of these microorganisms in processing plants of cattle and poultry slaughterhouses, and to characterize them with respect to pathogenicity potential, biofilm formation and antimicrobial resistance.

The study could isolate 14 *Listeria monocytogenes* and one *Salmonella* enterica from poultry slaughterhouses. *L. monocytogenes* puslotypes 5 and 9 were more frequently isolated than others. The authors conclude that these are persistent, biofilm forming strains that might constitute constant sources of contamination of processed meat.

**Comments**

The number of isolates recovered in this study are limited. The relevance of whole genome sequencing of the only isolate of *Salmonella* to this study is not clear. Although the isolates exhibited biofilm formation, this together with the frequency of isolation of two pulsotypes does not provide strong evidence to suggest that the isolates are persistent contaminants in these facilities. Further, the sources of contamination are obscure. The manuscript has abundantly used the available literature to support their findings. However, the results available from the study are too limited to make such conclusions.

**Major points**

- I am not convinced with the concept biofilm-induced contamination points, which is not defined in the manuscript. Past studies have analyzed the presence of *Listeria monocytogenes*, *Salmonella* etc from processing plants by swab analysis approach. In lines 303-304, you have cited studies from the same geographical region and similar sampling points. However, this manuscript considers swab isolates form surfaces as biofilm formers. (Lines 286-288: The present study is the first investigation of *L. monocytogenes* biofilms in poultry and cattle slaughterhouses facilities, equipment, and utensils in the region). Again in L289-290, it is stated that “The prior findings corroborate the present results”. Corroborate what? The presence of *L. monocytogenes* or the biofilm *L. monocytogenes*?
- The persistent contaminant from biofilms across different sampling time points and locations should be clearly defined.
- Table 2: Here, the number of swabs from different collection points are different. Please explain why.
- L307-308: Here, it is stated that *L. monocytogenes* was detected in two samples collected on different days and this could be due to persistent, biofilm-forming strains. How close were these sampling days?. If I am correct, 5 swabs yielded equal number of isolates. How many isolates were obtained from each positive sample? It is unlikely that you got just one isolate from a sample. If you got more than one isolate, did you compare them phenotypically and genetically and then select non-repetitive isolates?.
- Again in L308-309, it is stated that “*L. monocytogenes* was detected in two samples collected on different days, suggesting the presence of biofilm at the site”. Did you collect samples immediately after sanitization? How do you rule out recontamination after disinfection?
- L313-314: In Berrang et al. (2010), the source of L.monocytogenes was the raw meat. According to this study, a persistent drain subtype was identical with the raw meat subtype, and this clone was recovered frequently in their sampling. However, in your study, it appears that there is correlation between the source and the recovery of the clonal isolate from the meat chute.
- Which pulsotype was isolated frequently from meat chute? . Since you followed CDC protocol, you should be able to compare your PFGE pattern with those in PulseNet database.
- L440-442: Were L. monocytogenes resistant to chloramphenicol before the ban of the antibiotic? This is important to understand the effect of antibiotic ban on the resistance patterns.
- You have used 12^o^C to study biofilm formation. Does this simulate the temperature conditions of your sampling locations?
- Pulsotype 9 and 5 were more frequently isolated and probably the persistent strains as suggested in the manuscript. It is important to know if these pulsotypes exhibited higher biofilm formation compared to others which were not isolated frequently. This will strengthen your hypothesis that the persistence of these pulsotypes is due to their ability to form strong biofilms.
- The sections on antimicrobial susceptibility and biofilm formation by *Salmonella* Minnesota can be combined. The study could isolate a single Salmonella and the significance of this finding is very limited.
- Please avoid claiming “first time” in multiple places in the same manuscript, unless the finding is very significant.
- Materials and methods: Samples were collected from 3 poultry slaughterhouses and 2 cattle slaughterhouses. Eight visits were conducted in two facilities. If I got it correctly, it would be 8x3 =24 and 8x2-16 (40 in total). And in each visit, samples were collected from installations and the equipment. Were the same locations samples each time? If so, how many swabs from each of these? An illustration would be useful for the readers to understand your sampling strategy.
- L120: “…Biofilms were characterized..”. Biofilms can be formed on any of the above sampling locations (installations/equipment). Did you look at this possibility? Of did you select a few locations for biofilm bacteria?.

**Minor points**

- The abstract is too long, and you might consider shortening it.
- A Venn diagram can help understand the distribution of pulsotypes from different locations.
- I have indicated few minor corrections in the manuscript itself.
